# Malaria control and chemoprophylaxis policy in the Republic of Korea Armed Forces for the previous 20 years (1997–2016)

**DOI:** 10.1186/s12936-018-2449-4

**Published:** 2018-08-15

**Authors:** Jae Hyoung Im, Kyungmin Huh, Chang-Gyo Yoon, Hyeongtaek Woo, Jin-Soo Lee, Moon-Hyun Chung, Terry A. Klein, Jaehun Jung

**Affiliations:** 1 Department of Internal Medicine, Armed Forces Daejeon Hospital, Daejeon, Republic of Korea; 2 Department of Infectious Diseases, INHA University Hospital, Incheon, Republic of Korea; 3 Division of Infectious Diseases, Department of Medicine, Samsung Medical Center, Sungkyunkwan University School of Medicine, Seoul, Republic of Korea; 4 Department of Preventive Medicine, Armed Force Medical Command, Seongnam, Republic of Korea; 5 Department of Internal Medicine, Jeju University Hospital, Jeju, Republic of Korea; 6 Force Health Protection & Preventive Medicine, Medical Activity-Korea/65th Medical Brigade, Unit 15281, Apo, AP 96251 USA; 7 Department of Preventive Medicine, Korea University College of Medicine, Seoul, Republic of Korea

**Keywords:** Chemoprophylaxis, Hydroxychloroquine, Malaria, Military, *Plasmodium vivax*, Primaquine, Soldier

## Abstract

**Background:**

Vivax malaria reemerged along the Demilitarized Zone (DMZ), Republic of Korea (ROK), in 1993. While it was hypothesized that vivax malaria would spread throughout the peninsula, nearly all cases were due to exposure near the DMZ. To reduce spillover of vivax malaria to the civilian community, the ROK Ministry of National Defense (MND) initiated malaria prevention policies including a large-scale chemoprophylaxis programme in malaria high-risk areas in 1997. The present study investigated the overall changes in the incidence of malaria among ROK soldiers and the mass chemoprophylaxis program from 1997 to 2016.

**Results:**

Peak numbers of vivax malaria were reported in 2000, with most cases reported near the DMZ, before declining to the current levels. To combat the rapid increase in the number of malaria cases and its expansion throughout the ROK, the MND implemented mosquito control and personal protection programmes. The MND also implemented a large-scale vivax malaria chemoprophylaxis programme using hydroxychloroquine (400 mg weekly) in 1997, and primaquine (15 mg × 14 days) as terminal chemoprophylaxis in 2001. Additionally, an improved medical system enabled the rapid detection and treatment of malaria to reduce morbidity and decrease transmission of malaria from humans to mosquitoes. Following the full implementation of these programmes, the incidence of vivax malaria declined in both ROK Armed Forces and civilian populations. Subsequently, several changes in the ROK Armed Forces chemoprophylaxis programme were implemented, including the reduction of the period of hydroxychloroquine prophylaxis by 2 months (2008) and other changes in the chemoprophylaxis policy, e.g., only ROK Armed Forces personnel in moderate risk groups received terminal primaquine chemoprophylaxis (2011), and in 2016, the discontinuation of terminal primaquine chemoprophylaxis in moderate-risk area.

**Conclusions:**

The resurgence of vivax malaria in the ROK Armed Forces personnel near the DMZ was successfully suppressed through the implementation of a mass malaria chemoprophylaxis programme initiated by the MND in 1997, as well as several other factors that may have contributed to the reduction of malaria transmission since 2000. Given the current malaria situation in the ROK and North Korea, it is necessary to reevaluate the ROK Armed Forces and civilian malaria control policies.

**Electronic supplementary material:**

The online version of this article (10.1186/s12936-018-2449-4) contains supplementary material, which is available to authorized users.

## Background

**Fig. 1:**
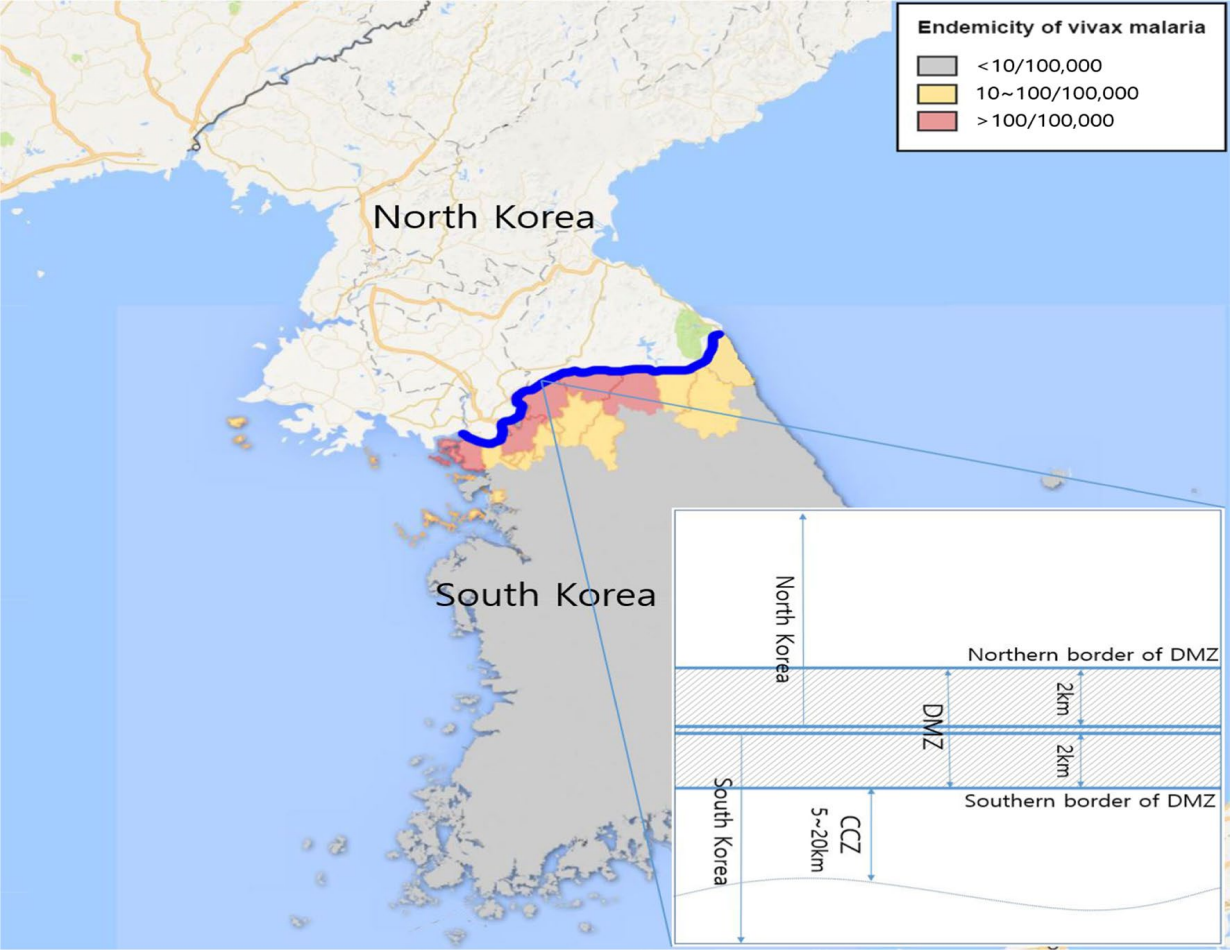
The demilitarized zone (DMZ) and distribution of vivax malaria (2016) in the Republic of Korea

**Fig. 2:**
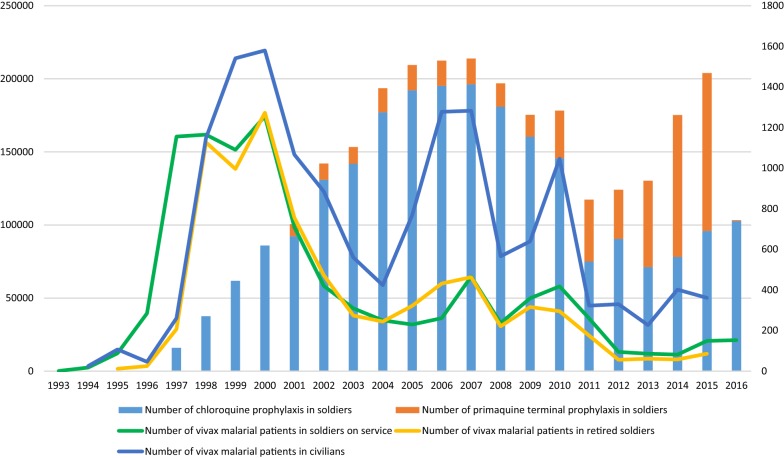
The annual number of ROK active duty soldiers administered hydroxychloroquine and primaquine chemoprophylaxis and the number of active duty soldiers, veterans, and civilians diagnosed with vivax malaria from 1993 to 2016

*Plasmodium vivax* was prevalent in Korea for many centuries [1, 2]. Following World War II and the separation of the Democratic People’s Republic of Korea (DPRK, North Korea) and the Republic of Korea (ROK, South Korea), the prevalence of vivax malaria gradually decreased, mostly due to passive malaria surveillance, rapidly growing economy with better housing, and better public health infrastructure. In 1979, the World Health Organization (WHO) declared the ROK to be malaria-free. However, *P. vivax* reemerged in the ROK in 1993 along the demilitarized zone (DMZ) [3], and rapidly increased to > 4000 vivax malaria cases by 2000 before declining (Fig. 1) [2]. By 2012, the number of vivax malaria cases declined to 555, possibly due to malaria control efforts (Fig. 2) [4], including a large-scale malaria chemoprophylaxis programme managed by the ROK Ministry of National Defense (MND).

Vivax malaria in the ROK Armed Forces has the following characteristics: (1) it is the only endemic malaria in the ROK, (2) it is not resistant to hydroxychloroquine (used by the ROK MND) or chloroquine, and (3) recurrence of malaria occurs in a portion of vivax malaria patients that have been administered 15 mg of primaquine for 14 days (terminal chemoprophylaxis and chemotherapy) [5, 6]. Moreover, male ROK soldiers are a relatively homogenous young group with low co-morbidities and demonstrate little immunity to malaria, in part, because most new recruits are from non-endemic areas. Approximately one-third of ROK soldiers are deployed and reside in moderate to high malaria-risk areas. Because they serve for 21–24 months, they experience two malaria-risk seasons from May through October during their compulsory period of service. Following their compulsory service, they remain in veteran status and receive medical reimbursement for diseases acquired during their service, including malaria.

The present study identified changes in malaria control policies, including chemoprophylaxis provided by the ROK MND to members of the ROK Armed Forces in moderate to high malaria risk areas over the past 20 years, vector control, institution of personal protection, and reduction in the number of malaria cases based on the modified policies.

## Methods

### Republic of Korea Armed Forces and DMZ

The ROK Armed Forces are composed of the Army, Navy (including Marines), and Air Force services. Members of the Air Force are distributed nationwide, but infrequently reside near the DMZ, while large numbers of Army and Navy personnel (particularly Marines) reside near or conduct operations inside the southern boundary of the DMZ, adjacent to the military demarcation line (MDL) that separates the DPRK and the ROK.

To understand the relationship between the ROK Armed Forces and vivax malaria, it is important to understand the DMZ environment. The DMZ serves as a military buffer zone that is 4 km wide and separated by the military demarcation line (MDL) into a southern (2 km) and northern (2 km) are that is 250 km long (Fig. 1). Although some ROK military police may be present inside the southern 2 km wide portion of the DMZ, in principle, it is considered a demilitarized area. Due to the sensitive status of the DMZ, military activities, except for human surveillance and mosquito control, are very limited. The Civilian Control Zone (CCZ) consists of a 5–20 km wide area that parallels the DMZ and is a safety zone where human activities are strictly regulated. Only a few designated citizens are permitted to reside in the CCZ, although many ROK soldiers reside or are deployed to the area for limited periods of time (Fig. 1). The reemergence of vivax malaria in the ROK in 1993 and its subsequent resurgence to high numbers of malaria cases in 2000 was considered, in part, to be due to the southward movement of malaria vectors belonging to the *Anopheles* Hyrcanus Group that can only be identified using polymerase chain reaction (PCR), from the southern boundary of the DPRK. Considering that vivax malaria risks are associated with the proximity of both military and civilian populations to the DPRK, where large numbers of malaria were consistently reported by WHO, the DMZ area poses a major vivax malaria risk area to the ROK Armed Forces. Evaluation of the travelling distances of *Anopheles* spp. (capture-mark-release-recapture) showed that they fly up to 25 km, with most being recaptured within a few kilometers [2]. Based on the assumption that malaria reemerged as a result of infected mosquitoes from the DPRK, ROK Armed Forces soldiers stationed near the DMZ were initially exposed to higher risks than civilian populations. Therefore, soldiers in the neighbouring areas of the DMZ (e.g., Ganghwa, Incheon, Goseong, Goyang, Dongducheon, Cheolwon, Hwacheon, Gimpo, Paju, Yeoncheon and Pocheon districts) were targeted for elimination of malaria, based on malaria risk factors (numbers of annual cases for the earlier years) (Fig. 1).

### Surveillance of patients with malaria

Data for ROK soldiers who received chemoprophylaxis from May 1997 to December 2016 were reviewed. Malaria cases were based on the MND infectious disease and Korean Centers for Disease Prevention and Control (KCDC) reporting systems. Patients were identified as positive for malaria based on a positive rapid malaria antigen test, detection of nucleic acid by polymerase chain reaction (PCR) of blood samples, or observation of malaria parasites in peripheral blood smears. Soldiers were divided into (1) those serving on active duty diagnosed with malaria and (2) veterans diagnosed with malaria < 2 years after they were discharged/retired from active duty, due to latent hepatic stage (hypnozoites) parasites. The reported incidence of malaria for recently discharged soldiers (< 2 years) was conducted by the infectious disease reporting system of the KCDC.

## Results

### Cases of vivax malaria for military personnel deployed/residing in or near the DMZ, since 1993

A ROK soldier residing near the DMZ was diagnosed with vivax malaria (blood smear) in 1993, following a hiatus of 14 years of the ROK being declared malaria-free by WHO. Thereafter, the number of vivax malaria cases, although sporadic, rapidly increased among ROK soldiers deployed/residing near the DMZ. Nationwide, by 1996, there were 356 malaria patients, including 310 soldiers (87.1%) and 46 civilians (12.9%). Due to the rapid spread of malaria along the DMZ and the potential for malaria to become a major threat throughout the ROK, the ROK Army established a malaria eradication policy. The policy included: filling low-lying areas that flooded (e.g., wheel ruts) and removal of vegetation from the borders of large areas of water for larval control, the use of spray pesticides and repellent/insecticide coils to control adult populations, and personal protective measures, e.g., installation of insect screens, mosquito nets, uniforms treated with permethrin, and application of repellents to exposed areas of the skin (Table 1). Mosquito repellents were infrequently used and were considered to be impractical because the supply per capita was very low due to the cost. In addition, larval mosquito control methods that included filling low-lying areas, tire ruts, and puddles and pesticide application for adult control were possible only outside the southern border of the DMZ, which was insufficient to control malaria vectors in the DMZ.

**Table 1:** Mosquito control and prevention policies developed by the ROK Armed Forces Medical Command

	Method	Contents	Notes
Larva	Filling of puddle	No use of organic insecticides	
	Cleaning of environment	Removal of empty bottles, cans, and other discarded containers	
Adult	Space spray application	2–3 times/week	
		10 km around the troop locations	
		20:00–21:00, 05:00–08:00	
		8 km/h vehicle speed, 1 km/h for portable sprayers	
	Residual spray application	1 time/week, no time limit	
		Window screens, walls, watch tower	
	Insect screens	From May to October	
	Mosquito bed nets	From May to October, treated with permethrin	
	Combat uniform treated with permethrin	From May to October, treated with permethrin, 1 times/month	Compliance: complete = 22%, partial 3.3%, none = 74.8% in 2007
	Repellent application	From May to October, diethylmetatolumide	Compliance: complete = 22.8%, partial 26.4%, none = 50.7% in 2007
	Repellent coil	From May to October	

### Start of the chemoprophylaxis by the Ministry of National Defense in 1997

Due to the rapid and remarkable increase of vivax malaria and the concern for the lack of effectiveness of the above-mentioned mosquito control and personal protective measure policies, in 1997 the MND ordered the combination use of hydroxychloroquine (blood stage) and primaquine (liver stage) to protect soldiers residing/deployed to malaria high-risk areas. Hydroxychloroquine (300 mg) was administered weekly, during the malaria transmission period (May–November), and primaquine (15 mg) was administered daily for 14 days, at the end of the malaria transmission season. A total of 15,981 ROK soldiers enrolled in the chemoprophylaxis programme in 1997, with numbers increasing to 86,010 by 2000 and 142,000 by 2003. While vivax malaria continued to increase in the ROK populations through 2000, the number of malaria cases among ROK active duty soldiers and recently veteran soldiers in 1998 and 1999, respectively. However, decreased numbers of malaria cases were not observed in civilian populations until 2001, after which there was a sharp decline in overall numbers of malaria patients. By 2004, there was a low of 826 cases reported among ROK active duty military, veterans, and civilians with increases/decreases through 2016 that were likely weather dependent (Fig. 2). These data indicated that the MND malaria chemoprophylaxis programme, that had been successful in the elimination of malaria during the 1970s, resulted in the reduction in the number of malaria cases among ROK military personnel, which was also relayed to the veteran and civilian populations.

Based on this information, the MND unified the treatment of malaria among ROK Army soldiers in an official document that recommended a regimen of hydroxychloroquine (600 mg initially, followed by 300 mg at 6 h, then 300 mg daily for 2 days) plus primaquine (15 mg × 14 days) for all malaria cases. During the malaria season, education programmes were implemented and public announcements were performed, so that suspected patients with chills, high fever, and sweats, were quickly transported to military hospitals for timely diagnosis and treatment to reduce morbidity and decrease man-mosquito malaria transmission. Additionally, the monetary compensation to recently retired soldiers, which included the cost of treatment, resulted in rapid treatment and increased reporting.

### Start of the primaquine terminal prophylaxis in 2001

The MND assessed the effectiveness of the chemoprophylaxis programme, and as a supplementary policy, the MND started primaquine terminal prophylaxis (15 mg primaquine once daily × 14 days), for soldiers that were discharged/retired during the malaria season. The programme started in 2001 with 8300 veterans receiving primaquine chemoprophylaxis, a number that increased to 16,500 in 2004. Among ROK malaria patients, the percentage of veteran soldiers with malaria reflected that of active duty soldiers, and decreased from 30.4% between 1998 and 2000 to 25.8% between 2002 and 2004. However, the percentage of recently discharged ROK soldiers diagnosed with malaria among all military personnel (active duty and recently discharged soldiers) did not change (49.2% between 1998–2000 and 50.3% between 2002–2004) (Additional file 1).

### Reduction of the duration of the hydroxychloroquine prophylaxis since 2008

Following an overall peak in the number of malaria cases among all ROK populations in 2000, the annual number of patients with malaria decreased to a low in 2004, with fluctuations observed through 2016, in part, due to weather patterns, e.g., moderate rains throughout the malaria season that resulted in increased mosquito populations or reduced rainfall that reduced larval habitat and adult populations. In part, due to the decreasing number of malaria cases, emphasis on malaria prevention decreased in military units, which resulted in lower compliance with the chemoprophylaxis programme (Additional file 1, Fig. 2). While compliance data are lacking for some years, hydroxychloroquine and primaquine compliance was similar for 2005–2007, 43.3–60.1% and 41.9–57.5%, respectively. Therefore, the MND reduced the period of chemoprophylaxis by 2 months, starting chemoprophylaxis in July instead of May.

In addition, MND quality control parameters for malaria diagnosis were difficult to determine because MND did not have sufficient numbers of experienced microscopists to examine peripheral blood smears. Therefore, since 2008, the MND supplied rapid malaria diagnostic kits (CareStart Malaria^®^, Accessbio), to the ROK Army Medical Corps, to reduce the time from first report to diagnosis. This reduced the time from the onset of the first paroxysm (chills, high fever and sweats) to diagnosis to an average of 30 h.

### Reduction in the number of individuals that received chemoprophylaxis since 2011

In addition to reducing the period of chemoprophylaxis (July to October) in 2008, the MND attempted to reduce the number of ROK soldiers placed on chemoprophylaxis. In 2011, ROK solders were categorized as deployed/residing in moderate- to high-risk areas based on the incidence of malaria during the previous year. ROK soldiers in malaria high-risk areas continued to receive chemoprophylaxis, while those in moderate-risk areas only received primaquine chemoprophylaxis at the end of the malaria season (October). Therefore, the number of ROK soldiers that received hydroxychloroquine/primaquine chemoprophylaxis decreased from 145,000 in 2010, to only 75,000 in 2011, while those placed on primaquine-only chemoprophylaxis increased from 22,000 in 2010, to 42,000 in 2011. However, similar numbers of malaria cases were reported among ROK soldiers and veterans following the implementation of the new policy (Additional file 1).

### Termination of primaquine prophylaxis in moderate-risk area, since 2016

In the 2010s, the number of soldiers with malaria in moderate-risk areas was significantly lower than before. The change resulted in concerns about the effectiveness of chemoprophylaxis in ROK soldiers of moderate-risk areas. Thus, in 2016, the MND ceased the primaquine-only prophylaxis for ROK soldiers in the moderate-risk areas, while the use of chloroquine-primaquine prophylaxis has continued in high-risk areas. It is still too early to assess the impact of the discontinuation of primaquine chemoprophylaxis in the moderate-risk area. In 2017, air conditioning was installed in soldier barracks, eliminating the need for open doors and unscreened windows for air circulation that reduced exposure to biting mosquitoes during the evening hours.

## Discussion

The number of malaria patients in the ROK military decreased following the implementation of the MND’s malaria control programme, which included a large-scale malaria chemoprophylaxis programme for soldiers residing in moderate- to-high-risk malaria areas. However, it is difficult to assess the absolute effectiveness of the programme without an understanding of the number of malaria cases among DPRK populations near the northern border of the DMZ, the vector competence of members of the *Anopheles* Hyrcanus Group and their distributions, vector populations and flight patterns, and the observed compliance among ROK soldiers that received chemoprophylaxis.

The origin of the reintroduction of vivax malaria in 1993 and the early outbreak was probably due to malaria-infected mosquitoes from the DPRK, which then spread from ROK military soldiers deployed/residing in/near the DMZ to nearby civilian populations [2, 7, 8]. Vivax malaria cases were reported more commonly among active duty and veteran ROK soldiers between 1993 (100%) and 2001 (49.8%); thereafter, however, civilian populations were more frequently reported with vivax malaria than military personnel. The early emergence of vivax malaria in the ROK military was primarily due to the proximity of ROK soldiers to the DMZ. As soldiers rotated from malaria high-risk areas to moderate-risk areas where civilians resided, they harbored latent liver stage parasites, developed malaria, and subsequently they infected mosquitoes that transmitted malaria to civilians (Fig. 2). With the implemention of the malaria chemoprophylaxis programme in 1997 and annual increases in numbers of ROK soldiers that received chemoprophylaxis, the number of reported malaria cases began to decrease in military populations in 1999, followed by decreased numbers of malaria cases among civilians. While the chemoprophylaxis programme continues to positively impact the incidence of malaria in ROK soldiers, civilians cannot afford medical care that provides long-term chemoprophylaxis, resulting in a higher number of cases than that observed in the ROK military and veteran groups, which provides a reservoir population for initiating malaria infections the following year and transmission of malaria throughout the malaria season.

The number of malaria cases among ROK soldiers that may have occurred if they did not use hydroxychloroquine-primaquine (CP) prophylaxis can be estimated, based on the number of malaria cases among civilians the following year. Although it is difficult to calculate the precise effectiveness of chemoprophylaxis, due to several factors, e.g., drug compliance, latency of vivax malaria (estimated to be 60–70%) [9] and the influence of malaria derived from the DPRK, there is a difference of about several hundred cases annually between the estimated incidence and the actual incidence of malaria among ROK soldiers. Differences have decreased gradually over time as lower numbers of malaria cases are reported, not only in the ROK, but also in the DPRK.

Since 2001, the number of patients with malaria decreased in both ROK civilians and soldiers, with annual fluctuations in all populations, most likely because of weather patterns, e.g., rainfall patterns that result in favorable or unfavorable *Anopheles* spp. larval breeding sites. This suggests that the malaria transmission patterns after 2000 were different from those before 2000 (DPRK → active duty soldiers → veterans within 2 years and civilians), and that autochthonous local transmission of malaria in the ROK can be considered as one of the primary causes. Furthermore, the decrease in civilian malaria cases since 2001 indicates that the incidence of malaria is also decreasing in groups that do not receive chemoprophylaxis, in part, due to reduced ROK Army malaria reservoir populations. Addtionally, other factors contribute to the reduction of malaria outbreaks.

First, there was improved rapid diagnosis that reduced the time from infection to diagnosis and period of infectivity to mosquitoes. By 2000, increased awareness of malaria contributed to decreased the period of time from the onset of symptoms to diagnosis and treatment compared to previous years. This was attributed, in part, to the promotion of government initiatives and the distribution of kits for rapid diagnosis [10]. In 2008, kits for rapid diagnostics became available to the ROK Army Medical Corps that further reduced the time required to diagnose malaria. Prior to 2008, the military documented the malaria incidence through various official letters, education and publication (The number of the MND documents on malaria, increased from 12 in 1997 to 538 in 2000), and the time to diagnosis was shortened in various ways, e.g., reducing the time from transfer of patients with fever from malaria endemic areas to military hospitals where patients were protected from biting mosquitoes.

Second, medical providers became more knowledgeable regarding malaria symptoms, diagnosis, and treatment protocols, including the use of primaquine early in the treatment to eliminate infective gametocytes that reduced man-mosquito malaria transmission [11, 12]. It is also possible to consider the role of hydroxychloroquine suppression in reducing the human malaria reservoir population. However, malaria patients that experienced a malaria paroxysm can be rapidly diagnosed by public health medical providers.

Third, there was likely a major decline in the number of vivax malaria cases reported in the DPRK from 2001 to 2008, in part, as a result of the ROK government providing the DPRK with anti-malarial drugs, medical equipment and contributed to training costs associated with early diagnosis and treatment (Additional file 1). This effort was likely responsible for the major decline in the number of malaria patients reported in the DPRK since 2003. In 2009, for political reasons the ROK government withdrew malaria control support, including the provision of anti-malarial drugs to the DPRK with subsequent increased numbers of malaria cases in the ROK between 2014 and 2016. The withdrawal of support to the DRPK, likely resulted in increased numbers of malaria cases in the DPRK and greater numbers of infected mosquitoes along the DMZ that spilled over to the ROK. Resumption of support to the DPRK in the future needs to be considered to produce further declines of malaria in both the DPRK and ROK populations.

Forth, mosquito control methods may have contributed to a reduction (undocumented) of malaria vectors, especially those that have higher vector potential capacity. Both the ROK military and civilian components initiated fogging operations during the early evening hours to reduce adult mosquito populations. While *Anopheles* spp. have been collected using Mosquito Magnet^®^ traps since 2005 near Panmunjeom by the U.S. military, the distribution of vector populations has not been determined due to the requirement for identification using PCR techniques [13, 14] (TA Klein, personal communication). However, a recent study by Chang et al. [15] showed that 69.5% of all *Anopheles* spp. collected at ROK military installations near the DMZ were *Anopheles kleini*, a primary malaria vector in the ROK, while *Anopheles sinensis*, a very poor vector of vivax malaria only accounted for 2.7% of all *Anopheles* spp. collected [16].

The number of malaria patients in the ROK Armed Forces began to decline 3 years before the number of soldiers that received chemoprophylaxis reached a maximum. Initially, soldiers that received chemoprophylaxis beginning in 2001 were deployed/resided in relatively malaria low-risk areas compared to the previous years, which suggests that chemoprophylaxis for ROK soldiers deployed/residing in malaria low-risk areas did not contribute significantly to the control of malaria in the ROK Armed Forces personnel. In addition, the malaria incidence in the high-risk groups was 7–9 times higher than that in the moderate-risk group. Thus, these results suggested that chemoprophylaxis for moderate-risk groups may not have significantly influenced the reduction of the incidence of malaria, while chemoprophylaxis for malaria high-risk groups likely affected the transmission of malaria in those areas. A review of the MND’s malaria control policies to reduce the number of soldiers on chemoprophylaxis requires continuous observation and evaluation to determine the effect of chemoprophylaxis on malaria transmission.

The large-scale chemoprophylaxis of the ROK Armed Forces is just one factor to be considered in the effort to control and eventually to eradicate malaria from the ROK, especially in view of the relatively low compliance. Low compliance may not only result in low prevention rates, but also influence other issues that make it difficult to access the effectiveness of the programme. Anti-malarial drug resistance should not be ignored [17]. Although a clinically significant level of chloroquine-resistant malaria has not yet been identified in the ROK, the F1076L variant of the *P. vivax* multidrug resistance 1 mdr-like gene (*pvmdr1*) has been reported [18]. This is a variant related to drug resistance in Thailand [19]. In addition, consideration should be given to side effects of the implementation of malaria chemoprophylaxis, e.g., although there is a low prevalence of glucose-6-phosphate dehydrogenase (G6PD) deficiency in ROK populations, hemolytic anemia, a potentially fatal adverse event, may occur in patients who are G6PD-deficient, when administered primaquine to treat the liver stage parasites to prevent the recurrence of vivax malaria [20]. Moreover, hydroxychloroquine can cause serious adverse effects, such as retinopathy, myopathy, and cardiopathy [21]. Finally, because vivax malaria is generally less severe than falciparum malaria, prevention of vivax malaria with anti-malarial agents has not been emphasized, unless access to medical facilities is difficult, e.g., during war or travel. The ROK Armed Forces possess tools for rapid diagnosis and relatively good accessibility to local medical clinics and hospitals. Considering these facts, the emphasis for chemoprophylaxis in Korean Armed Forces personnel seems to be waning, with a greater reliance on rapid diagnostics and treatment, especially when the incidence of vivax malaria is low.

There are some limitations in the present report, e.g., low compliance, which made it difficult to assess the exact effectiveness of the chemoprophylaxis programme established by the MND [22]. Because civilians are not allowed to reside in many of the malaria high-risk areas near the DMZ (including the CCZ), there are differences between the civilian residential areas and ROK Army installations where soldiers reside. Thus, it is difficult to make direct comparisons between civilian and soldier cohorts. In addition, some military data are restricted and cannot be reported in open source journals. However, the present study did not aim at obtaining accurate prevention rates of chemoprophylaxis, but at evaluating the overall direction and effectiveness of MND’s policy and annual vivax malaria trends.

## Conclusions

The expansion of the MND chemoprophylaxis that was initiated in 1997 was responsible, in part, for the reduction in the number of malaria cases among members of the ROK Armed Forces, observed in 1998. This effect carried over to the civilian populations where reduced numbers of annual malaria cases were observed since 2001. However, several factors other than chemoprophylaxis appears to have affected the reduction in the number of malaria cases since 2001, and it is necessary to re-establish malaria prevention/control policies according to the changing situation throughout all populations in the ROK, including the ROK Armed Forces. Thus, the cost and health benefits of chemoprophylaxis must be measured based on medication costs, morbidity/mortality, command emphasis (observed chemoprophylaxis), and the potential for vivax malaria parasites to develop tolerance/resistance to drugs.

## Additional file

**Additional file 1.** The number of ROK active duty soldiers provided primaquine malaria chemoprophylaxis, by year, and percent of active duty soldiers and veterans diagnosed with vivax malaria.

## Abbreviations

CCZ: Civilian Control Zone
DMZ: demilitarized zone
KCDC: Korea Centers for Disease Control and Prevention
MDL: Military Demarcation Line
MND: Ministry of National Defense
PCR: polymerase chain reaction
ROK: Republic of Korea
