# Supplementary material for: Malaria control and chemoprophylaxis policy in the Republic of Korea Armed Forces for the previous 20 years (1997–2016)
Source: Malar J. 2018 Aug 15;17:295. doi: 10.1186/s12936-018-2449-4 (PMC6094909; doi:10.1186/s12936-018-2449-4)
Supplement: Supplementary file 1 — Additional file 1. The number of ROK active duty soldiers provided primaquine malaria chemoprophylaxis, by year, and percent of active duty soldiers and veterans diagnosed with vivax malaria. [file 12936_2018_2449_MOESM1_ESM.docx]

Additional file 1. The number of ROK active duty soldiers provided primaquine malaria chemoprophylaxis, by year, and percent of active duty soldiers and veterans^1^ diagnosed with vivax malaria.

Variables

Without Primaquine

With primaquine

1998

1999

2000

2001^2^

2002

2003

2004

Primaquine prophylaxis numbers

━

━

━

8300

11050

11300

16500

Number of ROK veterans diagnosed with malaria

1127

996

1273

756

472

274

244

Percent malaria attributed to active duty soldiers and veterans

49.2%

47.7%

50.3%

51.6%

52.9%

46.9%

49.4%

Percent malaria attributed to veterans

32.8%

27.5%

31.0%

29.8%

26.6%

24.0%

26.6%

^1^ ROK veterans discharged/retired from active duty <2 years.
